# Early Prediction of Tumor Response to Neoadjuvant Chemotherapy and Clinical Outcome in Breast Cancer Using a Novel FDG-PET Parameter for Cancer Stem Cell Metabolism

**DOI:** 10.3390/jpm10030132

**Published:** 2020-09-17

**Authors:** Chanwoo Kim, Sang-Ah Han, Kyu Yeoun Won, Il Ki Hong, Deog Yoon Kim

**Affiliations:** 1 Department of Nuclear Medicine, Kyung Hee University Hospital at Gangdong, Kyung Hee University School of Medicine, Seoul 05278, Korea; 31073@hanmail.net; 2 Department of Surgery, Kyung Hee University Hospital at Gangdong, Kyung Hee University School of Medicine, Seoul 05278, Korea; drhsa.khmc@gmail.com; 3 Department of Pathology, Kyung Hee University Hospital at Gangdong, Kyung Hee University School of Medicine, Seoul 05278, Korea; wonki96@khu.ac.kr; 4 Department of Nuclear Medicine, Kyung Hee University Hospital, Kyung Hee University School of Medicine, Seoul 02447, Korea; hong.ilki@gmail.com

**Keywords:** cancer stem cell metabolism, breast cancer, neoadjuvant chemotherapy, FDG PET/CT

## Abstract

Cancer stem cells (CSCs) contribute to chemoresistance and tumor relapse. By using the distinct metabolic phenotype of CSC, we designed novel PET parameters for CSC metabolism and investigated their clinical values. Patients with breast cancer who underwent ^18^F-FDG PET/CT before neoadjuvant chemotherapy (NAC) were retrospectively included. We developed a method to measure CSC metabolism using standardized uptake value histogram data. The predictive value of novel CSC metabolic parameters for pathologic complete response (pCR) was assessed with multivariable logistic regression. The association between the CSC parameter and disease-free survival (DFS) was also determined. We identified 82 patients with HER2-positive/triple-negative subtypes and 38 patients with luminal tumors. After multivariable analysis, only metabolic tumor volume for CSC (MTVcsc) among metabolic parameters remained the independent predictor of pCR (OR, 0.12; *p* = 0.022). MTVcsc successfully predicted pathologic tumor response to NAC in HER2-positive/triple-negative subtypes (accuracy, 74%) but not in the luminal subtype (accuracy, 29%). MTVcsc was also predictive of DFS, with a 3-year DFS of 90% in the lower MTVcsc group (<1.75 cm^3^) versus 72% in the higher group (>1.75 cm^3^). A novel data-driven PET parameter for CSC metabolism provides early prediction of pCR after NAC and DFS in HER2-positive and triple-negative subtypes.

## 1. Introduction

Cancer cells within a tumor are heterogeneous with respect to metabolic phenotypes as well as genotypes. Historically, the clonal evolution model has explained that the tumor dynamics were derived from the serial accumulation of different driver mutations. However, the cancer stem cell (CSC) theory has also been providing a strong biologic basis for tumor heterogeneity even regardless of genetic backgrounds [1,2]. The CSCs, located at the apex of the tumor hierarchy, retain stemness features of self-renewal and differentiation potential. This minor subpopulation of cancer cells has now established a role in treatment failure [3]. In contrast to bulky differentiated cancer cells, CSCs can survive chemotherapy and radiotherapy; hence, these treatment-resistant cancer cells are responsible for tumor relapse as they are also implicated in the tumorigenesis [4].

Through oncogenic progression, tumors increase the complexity in cellular organization and gradually obtain the stem cell-like features, as cancer cells adapt to and interact with continuously changing tumor microenvironments [5]. Advanced tumors are thus comprised of cancer cells with varying levels of differentiation, consequently leading to cancer cell subsets exerting differential biological traits [5]. Recently, radiomics has been used to measure the intratumoral heterogeneity using texture analysis [6,7], but it has a major drawback. The results are not consistent across the studies [6,8]. The lack of reproducibility in radiomics modeling is primarily attributable to the radiomic features that cannot fully represent the true tumor characteristics. Therefore, new imaging parameters representative of central concepts within tumor biology urgently need to be developed.

The acquisition of a stem cell state in cancer cells is accompanied by metabolic reprogramming, resulting in a distinct metabolic phenotype of CSCs [9]. Normal stem cells are constitutively dependent on anaerobic glycolysis while differentiated cells preferentially rely on mitochondrial metabolism [10]. Likewise, proliferative CSCs enhance the glycolytic and shunt pathways to meet their metabolic requirements for energy, redox homeostasis, and building blocks, whereas differentiated cancer cells, which cannot divide any further, mainly depend on the mitochondrial oxidative phosphorylation [9,11]. Proliferative CSCs accordingly spend much more glucose than differentiated cancer cells do.

It was previously reported that poorly differentiated aggressive breast cancers showed upregulated expression of embryonic stem cell identity that is associated with Nanog, Oct4, Sox2, and c-Myc activation [12]. Similarly, another study found that high-grade breast cancers were enriched with a gene expression signature of mammary stem cells, while low-grade tumors had low stem cell content [13]. Recently, it was demonstrated that poorly differentiated tumors had significantly higher FDG uptake than well-differentiated tumors in various types of cancers [14]. The gene expression profiles of tumors with high versus low FDG uptake also revealed that embryonic stem-cell-related pathways were strongly associated with tumor glycolysis, a process termed “the Warburg effect” [14]. All these, collectively, suggest that enhanced glycolysis is the hallmark of CSC metabolism, which can be measured by ^18^F-FDG PET/CT scan.

Because ^18^F-FDG PET/CT visualizes the Warburg effect, selective CSC metabolism can be measured with standardized uptake value (SUV) histogram-based analysis within tumor voxels. Herein, we developed a new method to quantify the intratumoral heterogeneity by using the distinctive glucose metabolism between proliferative CSCs and differentiated cancer cells. We investigated the prognostic values of the novel PET parameters for CSC metabolism in breast cancer patients who received neoadjuvant chemotherapy (NAC). We hypothesized that the lower value of CSC metabolism would predict chemosensitivity as well as better clinical outcome.

## 2. Materials and Methods

### 2.1. Patients and Study Design

Patients with clinical anatomic stage II or III breast cancer from 2 centers were consecutively evaluated in this retrospective study between February 2012 and August 2019. We identified 127 eligible patients who underwent pretreatment ^18^F-FDG PET/CT and were treated with NAC. Patients received various NAC regimens: doxorubicin and docetaxel, doxorubicin and cyclophosphamide, or doxorubicin and cyclophosphamide followed by taxane. For the HER2-positive subtype, anti-HER2 agents were additionally applied in the neoadjuvant or adjuvant setting. Neoadjuvant HER2-targeted therapy regimens included trastuzumab only and pertuzumab combined with trastuzumab. Our institutional review boards approved this study (KHNMC 2020-01-017 and KHUH 2020-05-069), and informed consent was waived due to its retrospective nature. Clinicopathologic features and follow-up data were collected from the electronic medical records and pathology reports.

### 2.2. Histopathologic Analysis

Tumor histology and the molecular subtypes were determined by pretreatment core biopsies. Histologic grading was performed using the modified Scarff-Bloom-Richardson system. Estrogen receptor (ER) and progesterone receptor (PR) status were considered positive if at least 10% of tumor cells showed ER and PR expression. Low ER-positive tumors (1% ≤ ER < 10%) were considered negative because of their clinical behavior like ER-negative tumors [15]. HER2 overexpression was considered positive if tumor staining showed a 3+ pattern. If tumor staining was equivocal (2+ pattern), FISH was further used to confirm HER2 amplification. Ki-67 expression was considered high if there was positivity in ≥30% of the tumor cells. Tumor response to NAC was classified as pathologic complete response (pCR) when there was no residual invasive cancer in the breast and lymph nodes (LNs), although residual carcinoma in situ was allowed. The luminal subtype denotes ER-positive/HER2-negative tumors regardless of the Ki-67 index, while the HER2-positive subtype includes ER-positive/HER2-positive and ER-negative/HER-positive tumors.

### 2.3. ^18^F-FDG PET/CT Procedures

Before NAC initiation, all patients underwent baseline ^18^F-FDG PET/CT. Patients were required to fast at least 6 h before intravenous administration of 3.7 MBq/kg of ^18^F-FDG. Sixty minutes after the injection, a whole-body PET scan was performed by using a PET/CT scanner (Gemini TF; Philips Medical Systems, Cleveland, OH, USA). PET image reconstruction was done by CT-based attenuation correction with a voxel size of 4 × 4 × 4 mm. The two centers used the same PET/CT scanner with the same protocol. Conventional metabolic parameters including maximum SUV (SUVmax), metabolic tumor volume (MTV), and total lesion glycolysis (TLG) were measured. MTV and TLG were measured with a threshold of SUV 2.5. Volumetric parameters with a threshold of 40% of SUVmax were also measured: MTV40% and TLG40%.

### 2.4. Novel PET Parameters for CSC Metabolism

**Figure 1:**
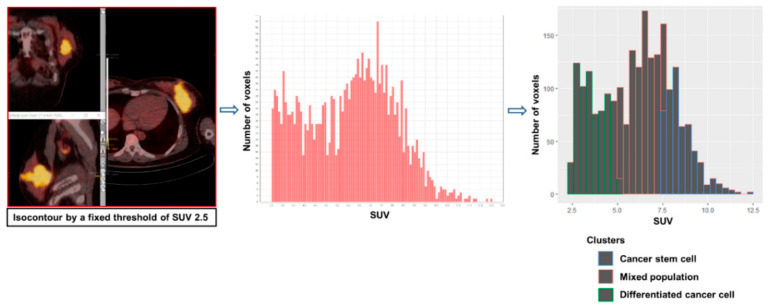
Workflow of measuring novel PET parameters for cancer stem cell metabolism.

**Figure 2:**
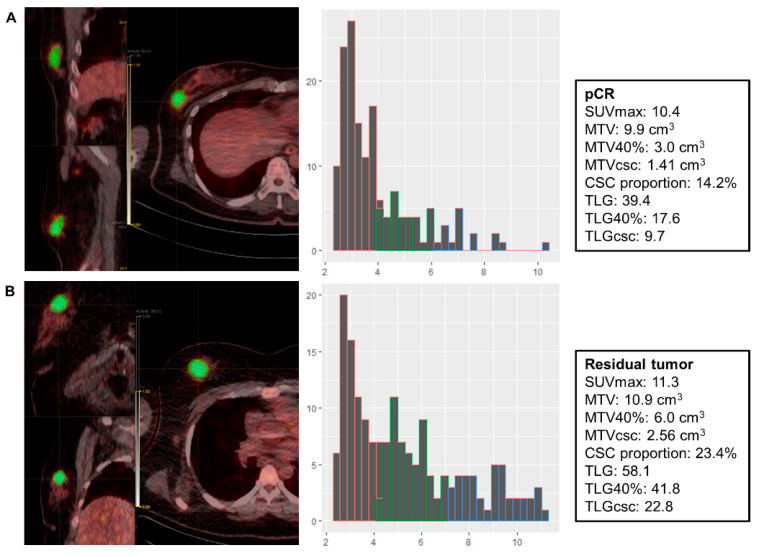
Representative cases of a patient with pCR and a patient with the residual tumor. (**A**) A patient with the triple negative subtype (cT2N1) achieved pCR after receiving AC + T #8. (**B**) A patient with the triple negative subtype (cT2N1) failed to achieve pCR after receiving AC + T #8. pCR: pathologic complete response; AC + T: doxorubicin and cyclophosphamide followed by paclitaxel; MTVcsc: metabolic tumor volume for cancer stem cell; TLGcsc: total lesion glycolysis for cancer stem cell.

To measure the novel parameters for CSC metabolism, a volume of interest encompassing the breast tumor or axillary metastatic LN was defined based on a fixed SUV threshold of 2.5. Patients with FDG non-avid tumors (SUVmax < 2.5) were excluded from the analysis. The SUV histogram data of the tumor voxels were extracted using LIFEx software (Ver. 5.10) [16]. The SUV of each voxel was grouped into 3 clusters by K-means clustering (K = 3) using R (Ver. 3.6.0). Because proliferative CSCs are more glycolytic than differentiated cancer cells, the volumetric parameters for the most glycolytic cluster were measured: MTV for CSC (MTVcsc) and TLG for CSC (TLGcsc). The least glycolytic cluster represents the metabolism of differentiated cancer cells, and the cluster with intermediate uptake indicates a mixed population of CSCs and differentiated cancer cells because we considered that there are voxels with the two phenotypes due to the limited spatial resolution of the PET image (4 × 4 × 4 mm). The CSC proportion of tumors was calculated by dividing MTVcsc by MTV. This whole process is depicted in Figure 1 and Figure 2.

### 2.5. Statistical Analysis

Multivariable logistic regression analysis was performed to evaluate the predictive values of clinicopathologic and metabolic parameters for pCR. Pathologic tumor response to NAC was predicted using the novel CSC parameter, and the prediction accuracy according to molecular subtypes was compared using the Pearson’s chi-square test. Disease-free survival (DFS) was calculated from the date of surgery to the date of tumor recurrence or the last follow-up date. Survival curves were plotted using the Kaplan-Meier method, and the association between the novel CSC parameter and DFS was examined by using the log rank test. All statistical analyses were conducted using SPSS Statistics (version 21, IBM Corporation, Armonk, NY, USA) for Windows. *p* values less than 0.05 were considered statistically significant.

## 3. Results

### 3.1. Patient Characteristics

**Figure 3:**
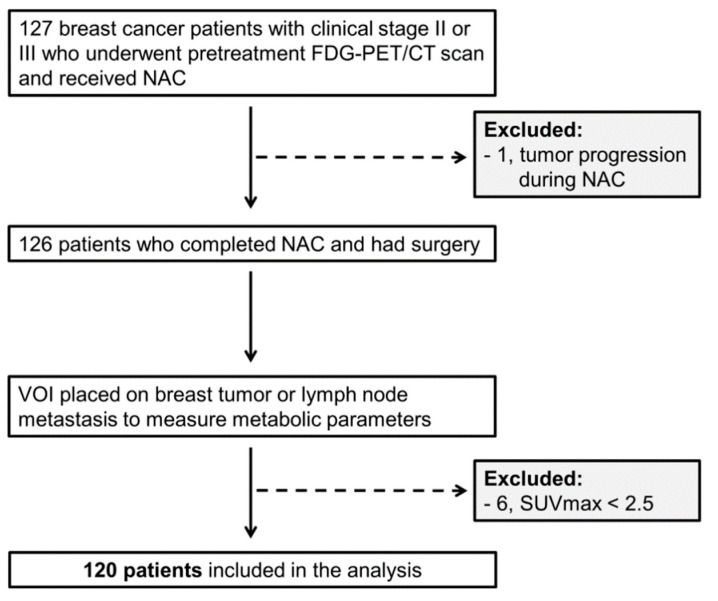
Patient flow. NAC: neoadjuvant chemotherapy; VOI: volume of interest; SUVmax: maximum standardized uptake value.

Among 127 eligible patients, 120 were included in the analysis (Figure 3). Patient and tumor characteristics are summarized in Table 1. pCR was achieved in 16 HER2-positive and six triple negative (TN) subtypes among 82 HER2-positive and TN subtypes. pCR was achieved in 16 patients out of 57 who received the anthracycline and taxane-based regimen. Six patients of 18 who were treated with the taxane-based regimen attained pCR, while none of the seven patients who received the anthracycline-based regimen achieved pCR. The pCR rate between the various NAC regimens was not significantly different (Fisher’s exact test; *p* = 0.279). Among 57 patients with HER2 overexpression/amplification, 31 patients received neoadjuvant anti-HER2 therapy. Twenty-two patients of them were treated with dual HER2 inhibition (trastuzumab + pertuzumab), and the remaining nine patients were treated with trastuzumab only. Of the 22 HER2-positive patients who were treated with dual HER2 inhibition, 10 patients achieved pCR. Of the 35 HER2-positive patients who were not treated with dual HER2 inhibition, only six patients achieved pCR (Pearson’s chi-square test; *p* = 0.021). Adjuvant endocrine therapy was applied to all ER-positive tumors. During follow-up, 16 patients experienced tumor relapse.

### 3.2. Association between the Achievement of pCR and Clinicopathologic/Metabolic Parameters in HER2-positive and TN Subtypes

The MTVcsc was measured in both breast tumors and LN metastases. The highest MTVcsc was measured in primary tumors in most cases (Table S1). Because none of 38 patients with the luminal subtype achieved pCR, the further analysis did not include this subtype. In HER2-positive and TN subtypes, the predictive values of several clinicopathologic and metabolic parameters for pCR were assessed in the logistic regression model (Table 2). In univariable analysis, the lower T stage, MTVcsc, TLGcsc, MTV, TLG, MTV40%, and TLG40% were significantly associated with pCR. Although the ER status showed marginal significance, it was included in the multivariable analysis since it is a known established predictor of pCR. In multivariable analysis, MTVcsc (OR of 0.12 per 1 cm^3^ increase in MTVcsc; *p* = 0.022) and ER negativity remained the independent predictors of pCR.

### 3.3. Prediction of the Pathologic Response with MTVcsc

Using the highest MTVcsc in the pCR group as a cutoff (<1.75 cm^3^), MTVcsc provided the opportunity to predict the pathologic tumor response to NAC with an accuracy of 74% (61/82) in HER2-positive and TN subtypes (Table 3). A more detailed analysis is provided in Table S2. In the luminal subtype, however, it showed a significantly lower accuracy of 29% (11/38) (Pearson’s chi-square test; *p* < 0.001). The data on prediction accuracy with MTVcsc in 82 HER2-positive and TN subtypes were further stratified according to the NAC regimens (Table 4), which revealed that the accuracy was similar across different NAC regimens. In the 57 HER2-positive tumors, however, the prediction accuracy was significantly higher in those who were treated with anti-HER2 NAC than those who were not (Pearson’s chi-square test; *p* < 0.007).

### 3.4. The Relation between MTVcsc and DFS in HER2-Positive and TN Subtypes

**Figure 4:**
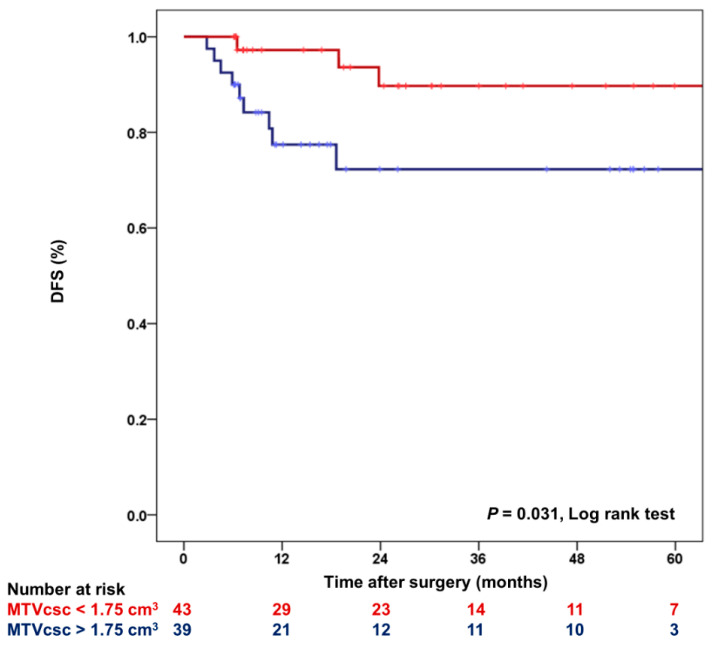
Kaplan-Meier plot for DFS according to MTVcsc in HER2-positive and TN subtypes. DFS: disease-free survival.

During a mean follow-up of 28.6 months (2.8–89.9 months), tumor recurrence in HER2-positive and TN subtypes was developed in 12 patients. All the 12 relapses occurred in the residual tumor group. With the same cutoff defined above, 3-year DFS was compared between the lower MTVcsc (<1.75 cm^3^) and the higher MTVcsc (>1.75 cm^3^) groups (Figure 4). Three-year DFS was significantly higher (*p* = 0.031) in the lower MTVcsc group (90%; 95% CI, 78–100%) than the higher MTVcsc group (72%; 95% CI, 56–89%).

## 4. Discussion

In the present study, we designed the novel metabolic parameter for proliferative CSC, and this is the first attempt to selectively measure the CSC metabolism from ^18^F-FDG PET/CT. Previously, the prognosis for breast cancer was predicted using CSC markers such as ALDH1 and CD44+/CD24− [17,18]. While insightful, their clinical implication is hampered by variable expression and non-specific natures [18,19]. Furthermore, the immunohistochemical staining of biopsy specimens cannot characterize the whole tumor because tumors exhibit spatial heterogeneity. These limitations underscore the role of ^18^F-FDG PET/CT as a non-invasive whole-body in-vivo imaging, depicting the tumor as a whole.

Since tumor-initiating cells were first identified in solid tumors in the early 2000s [20], mounting evidence suggests that tumor proliferation is driven by the metabolic reprogramming of CSCs that are a molecularly distinct subpopulation within a tumor [3,4,9]. The metabolic demand for proliferative CSCs is high because tumor proliferation requires the synthesis of major macromolecules, and glucose is the major nutrient for the anabolic growth supporting glycolysis, the pentose phosphate pathway, and the TCA cycle [21]. Therefore, the glucose requirement for proliferative CSCs is much higher than differentiated cancer cells that are least likely to divide any further [3,22]. Indeed, poorly differentiated tumors exhibited significantly higher SUVmax than well-differentiated tumors on ^18^F-FDG PET/CT across several types of cancers [14], and poorly differentiated or high-grade breast cancers were enriched for cancer stemness than well-differentiated or low-grade breast cancers [12,13].

CSCs possess intrinsic chemoresistance mediated by several pathways, some of which are induced by hypoxia-inducible factors (HIFs) [23,24]. Cancer cells are coping with the hypoxic tumor microenvironment where HIFs are stabilized, which in turn diverts metabolic pathways in favor of glycolysis [25,26]. HIFs facilitate the metabolic switch to enhanced glycolysis by inhibiting pyruvate from entering mitochondria [25,26]. Additionally, hypoxia plays a crucial role in promoting a CSC phenotype by increasing the expression of c-Myc, Oct4, Wnt, and Notch, which confer cancer stemness in an HIFs-dependent manner [24,27]. Therefore, the hypoxia signaling pathway is central to upregulated glycolysis in CSCs [26,28].

All these findings led us to build a new method to distinguish the CSC metabolism of tumors by exploiting histogram data from ^18^F-FDG PET/CT. Because proliferative CSCs are more glycolytic than their differentiated progeny, which makes up the bulk of the tumor [11,26,28,29,30], cancer cells with varying degrees of differentiation within a tumor exhibit different levels of glycolysis, which should be reflected in an SUV histogram. Previously, a similar approach was applied to breast cancer to analyze intratumoral heterogeneity, in which the cluster with the highest FDG uptake correlated with the highly aggressive phenotype while the cluster with lower FDG uptake corresponded to the less aggressive area [31]. The results of this study demonstrate that MTVcsc from a single pretreatment ^18^F-FDG PET/CT can predict whether the breast cancer patient would respond to NAC and experience a relapse after completion of treatment, especially in HER2-positive and TN subtypes.

NAC is currently the standard treatment for large or locally advanced breast cancer. With this approach, it offers opportunities for downsizing the tumor and breast conservation. Moreover, patients with pCR have survival benefits compared with patients who have residual tumors after NAC [32]. However, a recent meta-analysis that compared the clinical outcomes between neoadjuvant and adjuvant chemotherapies revealed that NAC did not show a survival advantage over an adjuvant setting [33]. Rather, NAC was associated with higher local recurrence after breast-conserving surgery [33]. Hence, it is critical to predict early who would be more likely to attain pCR. ER negativity is an established predictor of pCR, but approximately half of ER-negative tumors still cannot achieve pCR to NAC. Predicting pCR with ^18^F-FDG PET/CT in breast cancer has been intensively studied, and, currently, the most powerful predictor from this modality is using the change in tumor SUVmax after 1 or 2 cycles of NAC in HER2-positive and TN tumors [34,35,36,37]. This strategy reported a prediction accuracy of 75–80% [34,35,36], which is comparable to the present study. It was implemented to decide to add bevacizumab to predicted non-responders in a clinical trial and was found to be helpful in NAC optimization [38]. Nevertheless, this approach requires at least two PET/CT scans to be done, which is both time and cost consuming. In this regard, the present study outperforms the previous reports by presenting promising results about the prediction of pCR as well as prognosis at initial staging. Additionally, clinical trials of selectively not performing breast surgery in patients who achieve pCR are ongoing, as concerns about no benefit over surgery are being raised in such patients [39]. Consequently, a good surrogate biomarker of patient selection for optimal neoadjuvant chemotherapy is indeed needed.

The lower MTVcsc was also predictive of better survival in HER2-positive and TN subtypes. Patients with the lower MTVcsc experienced fewer tumor relapses with significantly higher DFS than patients with the higher MTVcsc. Besides, none of the patients with the higher MTVcsc achieved pCR after NAC. Although residual tumors were all removed by surgery, residual tumor cells might have entered circulation before surgery because chemotherapeutic stress can induce epithelial to mesenchymal transition that confers tumor cells with invasive motility [40]. Notably, it was reported that residual breast cancers surviving standard treatments were enriched for CSCs with a mesenchymal phenotype [41,42]. Consequently, therapy-resistant cancer cells that remain after NAC are likely to spread to distant organs and regrow afterward. Tumors with the higher MTVcsc that hardly achieve pCR are thus more likely to experience tumor recurrence.

Intriguingly, the prediction accuracy of treatment response using MTVcsc varied according to the molecular subtypes (Table 3 and Table S2). In contrast to HER2-positive and TN subtypes, the lower MTVcsc could not successfully predict the pathologic response to NAC in luminal tumors, which is probably the reflection of the intrinsic difference between these molecular subtypes. In ER-positive breast cancer where late recurrence is prominent, disseminated cancer cells remain dormant during the latent period, and this metastatic dormancy is attributable to quiescent CSCs [43]. Breast CSCs display two distinct phenotypes, in which epithelial-like CSCs are proliferative, whereas mesenchymal-like CSCs are quiescent [44,45]. The metabolic demand for quiescent CSCs is even lower than differentiated cancer cells because these cells are in a dormant state [45,46]. In stark contrast, proliferative CSCs increase their metabolic demand by upregulating both glycolysis and oxidative phosphorylation [46]. Although luminal tumors had lower MTVcsc in more than half of the cases, they could not achieve pCR in most cases. This is possibly contributed to their propensity to be in a quiescent CSC state since tumor dormancy was demonstrated to be associated with luminal differentiation of breast cancer [47,48]. The CSC plasticity between the two phenotypes imposes a therapeutic challenge, with each state exhibiting drug resistance by a separate mechanism [45]. The quiescent CSCs harbor inherent chemoresistance because most cytotoxic agents mainly target rapidly dividing cancer cells. Dynamic tumor repopulation during chemotherapy revealed characteristic responses of the two CSC phenotypes [49]. While proliferative CSCs showed a modest sensitivity to chemotherapeutic insults, quiescent CSCs went through a phenotypic transition to the proliferative state in response to mitogens released from nearby chemosensitive cancer cells [49,50]. In this context, it is postulated that luminal subtypes might prefer CSCs in a quiescent state, which exhibits a less glycolytic feature, and MTVcsc represents the glucose metabolism of proliferative CSCs.

This study has some limitations. Firstly, due to retrospective design, the treatment used in NAC was diverse across patients. Different NAC regimens can have an impact on the efficacy of pCR. Especially in the HER2-positive subtype, dual inhibition of HER2 by combining trastuzumab with pertuzumab significantly improves the pCR rate. Due to the limited number of patients with HER2-positive and TN subtypes, respective analysis in these subtypes could not be performed as well. The higher cutoff value of MTVcsc in predicting pCR is expected in the HER2-positive subtype than the TN subtype because HER2 signaling is an effective regulator for CSC maintenance, as evidenced by a decrease in CSC proportion resulted from HER2 blockade [44,51]. Thus, a separate analysis in each subtype on a large scale will provide further insights into CSC properties associated with molecular subtypes. In addition, although the novel method to measure CSC metabolism was based on the established tumor biology, the approach is rather speculative in that it is still lacking in direct evidence for CSC identification. Further studies investigating the correlation between MTVcsc and the cancer stemness index [52] are needed to strengthen our results. Lastly, we excluded FDG non-avid tumors from the analysis. The analysis was not applicable in the tumors with SUVmax < 2.5 because they could not be defined on PET images. Six FDG non-avid tumors were excluded, and they were all ER-positive tumors.

## 5. Conclusions

In conclusion, the novel data-driven PET parameter for CSC metabolism in the proliferative phenotype was successfully designed based on biologic features central to tumor heterogeneity. The MTVcsc can be a biomarker in early predicting pCR to NAC and DFS in HER2-positive and TN subtypes. Given the prognostic value of achieving pCR, treatment optimization could be initially guided because MTVcsc might determine who would benefit from NAC.

## Figures and Tables

**Table 1:** Patient and tumor characteristics.

Characteristics	*N* = 120
Age (years), median (range)	49 (25–72)
Histology	
IDC	115 (96%)
ILC/other	5 (4%)
Clinical T stage	
T1-2	79 (66%)
T3-4	41 (34%)
Clinical anatomic stage	
IIA-IIIA	97 (81%)
IIIB-IIIC	23 (19%)
ER status	
Positive	65 (54%)
Negative	55 (46%)
Molecular subtype	
Luminal (HER2-negative)	38 (32%)
HER2-positive	57 (47%)
Triple negative	25 (21%)
NAC regimen	
Anthracycline and taxane based	92 (77%)
Anthracycline based	10 (8%)
Taxane based	18 (15%)
Anti-HER2 therapy in 57 HER2-positive patients	
Neoadjuvant	31 (55%)
Adjuvant only	19 (33%)
None	7 (12%)
NAC response	
pCR (ypT0/is ypN0)	22 (18%)
Residual tumor	98 (82%)
Surgery	
Breast-conserving surgery	66 (55%)
Mastectomy	54 (45%)
Recurrence	
Yes	16 (13%)
No	104 (87%)

Values are *n* (%) unless otherwise specified. IDC: invasive ductal carcinoma; ILC: invasive lobular carcinoma; ER: estrogen receptor; NAC: neoadjuvant chemotherapy; pCR: pathologic complete response.

**Table 2:** Univariable and multivariable logistic regression analysis of predictive factors for pCR in HER2-positive and triple negative (TN) subtypes.

Parameters	HER2-Positive/TN		Univariable Analysis			Multivariable Analysis		
	pCR (*N* = 22)	Residual Tumor (*N* = 60)	OR	95% CI	*p* Value	OR	95% CI	*p* Value
T stage								
1–2	20 (38%)	33 (62%)	8.18	1.75–38.16	**0.007**			
3–4	2 (7%)	27 (93%)	1.00					
Clinical anatomic stage								
IIA-IIIA	20 (30%)	46 (70%)	3.04	0.63–14.66	0.165			
IIIB-IIIC	2 (12%)	14 (88%)	1.00					
Histologic grade								
1-2	8 (21%)	30 (79%)	1.00					
3	11 (28%)	28 (72%)	1.47	0.52–4.19	0.468			
Missing	3	2						
Ki-67								
Low, <30%	8 (24%)	25 (76%)	1.00					
High, ≥30%	9 (20%)	35 (80%)	0.80	0.27–2.37	0.692			
Missing	5	0						
ER status								
Positive	4 (15%)	23 (85%)	1.00			1.00		
Negative	18 (33%)	37 (67%)	2.80	0.84–9.31	0.093	8.37	1.75–40.1	**0.008**
Metabolic parameters ^a^								
MTVcsc (cm^3^)	0.9 (0.3–1.7)	2.8 (0.1–38.0)	0.37	0.19–0.72	**0.003**	0.12	0.02–0.74	**0.022**
TLGcsc	5.0 (1.4–38.5)	19.5 (0.2–593.4)	0.92	0.86–0.98	**0.010**			
CSC proportion (%)	15.9 (6.1–31.8)	21.5 (5.6–39.1)	0.94	0.87–1.00	0.068			
SUVmax	6.3 (3.3–28.7)	8.2 (3.4–23.7)	0.93	0.82–1.06	0.271			
MTV (cm^3^)	4.6 (1.7–13.8)	13.3 (1.2–170.7)	0.85	0.76–0.95	**0.005**			
TLG	17.1 (4.7–111.3)	57.6 (3.3–1386.9)	0.98	0.96–0.99	**0.011**			
MTV40% (cm^3^)	3.6 (1.7–13.8)	9.1 (1.2–90.3)	0.78	0.65–0.93	**0.005**			
TLG40%	16.0 (4.7–66.3)	47.0 (3.3–1006.1)	0.96	0.94–0.99	**0.008**			

^a^ Continuous variables, values are median (range). Bold values are statistically significant. TN: triple negative; OR: odds ratio; CI: confidence interval; MTVcsc: metabolic tumor volume for cancer stem cell; TLGcsc: total lesion glycolysis for cancer stem cell.

**Table 3:** Prediction of tumor response to NAC using MTVcsc in HER2-positive/TN vs. luminal subtypes.

	HER2-Positive/TN (*N* = 82)		Luminal (*N* = 38)	
	pCR (*N* = 22)	Residual Tumor (*N* = 60)	pCR (*N* = 0)	Residual Tumor (*N* = 38)
MTVcsc < 1.75 cm^3^	22	21	0	27
MTVcsc > 1.75 cm^3^	0	39	0	11
Prediction accuracy	74% (61/82)		29% (11/38)	

**Table 4:** Prediction of tumor response to NAC using MTVcsc in HER2-positive and TN subtypes with various NAC regimens.

	**Anthracycline and Taxane** **Based (*N* = 57)**		**Taxane** **Based (*N* = 18)**		**Anthracycline** **Based (*N* = 7)**	
	**pCR (*N* = 16)**	**Residual** **Tumor (*N* = 41)**	**pCR (*N* = 6)**	**Residual** **Tumor (*N* = 12)**	**pCR (*N* = 0)**	**Residual** **Tumor (*N* = 7)**
MTVcsc < 1.75 cm^3^	16	14	6	5	0	2
MTVcsc > 1.75 cm^3^	0	27	0	7	0	5
Prediction accuracy	75% (43/57)		72% (13/18)		71% (5/7)	
	**with Anti-HER2** **NAC (*N* = 31)**		**without Anti-HER2** **NAC (*N* = 26)**			
	**pCR (*N* = 12)**	**Residual** **Tumor (*N* = 19)**	**pCR (*N* = 4)**	**Residual** **Tumor (*N* = 22)**		
MTVcsc < 1.75 cm^3^	12	5	4	13		
MTVcsc > 1.75 cm^3^	0	14	0	9		
Prediction accuracy	84% (26/31)		50% (13/26)			

## Supplementary Materials

The following are available online at https://www.mdpi.com/2075-4426/10/3/132/s1, Table S1: MTVcsc measurement in target lesions, Table S2: Prediction of tumor response to NAC using MTVcsc according to the molecular subtypes.
