# Supplementary material for: Early Prediction of Tumor Response to Neoadjuvant Chemotherapy and Clinical Outcome in Breast Cancer Using a Novel FDG-PET Parameter for Cancer Stem Cell Metabolism
Source: J Pers Med. 2020 Sep 17;10(3):132. doi: 10.3390/jpm10030132 (PMC7565130; doi:10.3390/jpm10030132)
Supplement: Supplementary file 1 [file jpm-10-00132-s001.pdf]

Supplementary Materials

Table S1. MTVcsc measurement in target lesions.

Highest MTVcsc	**HER2-positive/TN (*N*=82)**		**Luminal (*N*=38)**	
	pCR (*N*=22)	Residual tumor (*N*=60)	pCR (*N*=0)	Residual tumor (*N*=38)
Breast tumor	18	57	0	33
Lymph node metastasis	4	3	0	5

Table S2. Prediction of tumor response to NAC using MTVcsc according to the molecular subtypes.

	**TN (*N*=25)**		**HER2-positive/ non-luminal (*N*=30)**		**Luminal B (*N*=27)**	
	pCR (*N*=6)	Residual tumor (*N*=19)	pCR (*N*=12)	Residual tumor (*N*=19)	pCR (*N*=4)	Residual tumor (*N*=23)
MTVcsc < 1.75 cm^3	6	3	12	5	4	13
MTVcsc > 1.75 cm^3	0	16	0	13	0	10
Prediction accuracy	88% (22/25)		83% (25/30)		52% (14/27)	
